# Excessive or reduced rest-day sleep compensation linked to depression in Chinese adults

**DOI:** 10.3389/fpsyt.2025.1601613

**Published:** 2025-07-04

**Authors:** Yanliqing Song, Long Wang, Yue Liu

**Affiliations:** ^1^ College of Sports, Nanjing Tech University, Nanjing, China; ^2^ School of Athletic Performance, Shanghai University of Sport, Shanghai, China

**Keywords:** sleep compensation, depression, pandemic period, mental health, public health intervention

## Abstract

**Background:**

This study aims to evaluate the impact of Rest Day Catch-up Sleep (RDCS) patterns on depression among Chinese adults during the pandemic and to explore the relationship between different levels of compensation and the likelihood of depression.

**Methods:**

This study included 3,981 participants, who were divided into five groups based on changes in rest day sleep duration: no change in sleep duration (RDCS = 0 h), reduced sleep duration (RDCS < 0), moderate catch-up sleep (1 h < RDCS < 2 h), and long catch-up sleep (RDCS ≥ 2 h). A multivariable logistic regression model was used to analyze the relationship between RDCS and depression. Stratified logistic regression and interaction effect analyses were conducted to explore demographic differences in the association between RDCS and depression.

**Results:**

In the fully adjusted model, participants with reduced sleep duration had an odds ratio (OR) of 2.12 (95%CI [1.31 – 3.46]) for depression, while those with long catch-up sleep had an OR of 1.60 (95%CI [1.29 – 1.98]). Stratified logistic regression and interaction effect analyses indicated that the association between RDCS < 0 h and depression was more significant among individuals with a Body Mass Index (BMI) < 25 kg/m², while the association between RDCS ≥ 2 h and depression was more significant among individuals with a general self-rated health status.

**Conclusion:**

The results of this study indicate that both reduced sleep duration and excessive catch-up sleep during the pandemic are associated with an increased likelihood of depression. These findings highlight the importance of maintaining stable sleep patterns during special periods and provide scientific evidence for the development of targeted public health interventions.

## 1 Introduction

Depression is one of the most prevalent mental health disorders worldwide, affecting approximately 300 million people and ranking among the top three causes of disability (1, 2). Its burden is substantial not only in middle-aged and elderly populations but also among young adults (3). The increasing incidence of depression has severe public health implications, impacting quality of life, work productivity, and social functioning.

Recent studies emphasize that sleep disturbances are a modifiable risk factor for depression, with strong associations between shortened sleep duration, disrupted sleep continuity, and the onset of depressive symptoms (4). Given that sleep issues are both a symptom and a contributor to depression (5), understanding how sleep regulation interacts with mental health outcomes is crucial. Previous findings indicate that both short (≤6 hours) and long (≥9 hours) sleep durations can elevate depression risk (6, 7), underscoring the importance of maintaining optimal sleep balance.

Sleep deprivation, particularly insufficient sleep during workdays, has emerged as a major public health concern. Modern lifestyles, characterized by work pressure, environmental disruptions, and social obligations, often prevent individuals from obtaining adequate sleep (8). Such sleep deficits can trigger physiological and psychological stress, making individuals more susceptible to depression, anxiety, and cognitive decline (9).

A key phenomenon observed in response to weekday sleep insufficiency is Rest Day Catch-up Sleep (RDCS) —a compensatory behavior where individuals extend sleep duration on rest days to recover from prior sleep deficits (10). While RDCS is commonly practiced to counteract sleep debt, its long-term effects on mental health, particularly depression risk, remain unclear. Existing research suggests that RDCS has positive effects on cardiovascular health and quality of life (11, 12), but whether it serves as an effective psychological recovery mechanism or introduces adverse effects through circadian misalignment remains inconclusive.

In contemporary society, weekend sleep patterns tend to deviate significantly due to two opposing phenomena: (1)Weekend Sleep Deprivation – caused by social jet lag, where individuals delay sleep onset due to social engagements, leading to circadian misalignment and increased mental fatigue. (2)RDCS – an effort to compensate for weekday sleep deficits, typically characterized by prolonged sleep duration, which may or may not result in effective psychological recovery.

Both phenomena can contribute to disrupted sleep homeostasis, potentially exacerbating depressive symptoms. While short-term RDCS may provide temporary relief, excessive compensation (>2 hours) may interfere with circadian rhythms, reducing sleep efficiency and worsening mood stability. The precise balance between sleep deprivation, compensatory sleep, and depression risk requires further empirical investigation.

Since late 2019, the COVID-19 pandemic has led to global disruptions in daily routines, profoundly affecting both physical and mental health. Strict lockdowns, social isolation, and economic uncertainties have significantly exacerbated depression rates worldwide (13). The pandemic contributed to a 27.6% increase in major depression cases, translating to an excess of 52.3 million cases globally (14).

Moreover, the pandemic drastically altered sleep habits, leading to higher prevalence of sleep disorders. A meta-analysis of 250 studies (≈500,000 participants) estimated that 40% of individuals experienced pandemic-related sleep disturbances, independent of external variables (15). In this context, understanding the impact of RDCS on depression becomes particularly critical, as individuals relied on sleep compensation mechanisms to manage mental stress during extended lockdowns.

This study aims to evaluate the relationship between RDCS patterns and depression, particularly during the pandemic. It seeks to determine whether compensatory sleep behaviors effectively mitigate depressive stress or introduce negative consequences through overcompensation. Investigating these mechanisms will provide scientific insights into the interactions between sleep regulation and mental health, informing targeted public health interventions to enhance psychological resilience in crisis periods.

## 2 Methods

### 2.1 Study population

The China Family Panel Studies (CFPS) is a nationwide comprehensive project conducted biennially. It covers three levels: community, family, and individual, collecting data on household income and expenditure, asset status, individual psychological and physiological conditions, utilization of health services, medical expenses, and more. This detailed data collection provides a comprehensive depiction of the evolution of Chinese society in areas such as socioeconomics, demographics, education, and health. The latest CFPS data, released in 2020, was meticulously collected using advanced computer-assisted survey techniques. The baseline sample covers 25 provinces (autonomous regions and municipalities), ensuring broad coverage and good representativeness. However, publicly available information does not specify the exact data collection timeframe.

The CFPS project strictly adheres to research ethical standards and has received formal authorization from the Biomedical Ethics Committee of Peking University (Ethics Approval Number: IRB00001052-14010). Additionally, the research team obtained explicit informed consent from participants before the survey commenced, ensuring ethical compliance and the confidentiality of participant information.

During the sample preparation phase, we applied consistent and rigorous standards, excluding records with missing key variables, including demographic characteristics and the Center for Epidemiologic Studies Depression (CES-D) Scale scores. This screening process not only ensured the integrity of the dataset but also provided a solid foundation for subsequent statistical analyses. After this process, we finalized 3,981 samples for inclusion in this study.

To ensure data precision and replicability, this study adopted strict inclusion and exclusion criteria to refine the sample selection process.

Participants were eligible for inclusion if they were between the ages of 18 and 65, allowing for a comprehensive examination of adult sleep behavior variations. Additionally, only individuals who completed both sleep pattern assessments and the depression questionnaire based on the CES-D were retained to ensure the validity of response data. Furthermore, the study required participants to have complete demographic and socioeconomic information, enabling appropriate statistical adjustments and minimizing the risk of confounding variables.

Conversely, exclusion criteria were applied to maintain the integrity of the analysis. Individuals diagnosed with chronic sleep disorders, such as insomnia or sleep apnea, were excluded to prevent baseline sleep conditions from interfering with the study’s findings. Similarly, respondents with a history of psychiatric disorders, including clinically diagnosed depression or bipolar disorder, were removed to uphold the validity of depressive symptom analysis. Moreover, extreme sleep duration cases, defined as individuals sleeping less than 3 hours or more than 12 hours per night, were eliminated to enhance the stability of statistical models and reduce the likelihood of outlier effects.

This rigorous selection process ensured that the study focused on a representative sample, allowing for more reliable conclusions regarding the relationship between RDCS and depressive symptoms.

It is important to note that the CFPS 2020 data collection took place during the COVID-19 pandemic, a period characterized by remote work, social isolation, and psychological stress, which may independently affect sleep patterns and depression symptoms. However, the dataset does not include employment status, detailed socioeconomic conditions, or household structure, limiting our ability to directly control for these pandemic-related factors.

### 2.2 Assessments of depression

Assessment of Depression: The CESD-8 scale, a simplified version of the CES-D Scale, was developed by Linda J. Radloff, a researcher at the National Institute of Mental Health (16). This scale retains the core concepts and structure of the original scale and is used for the rapid assessment of the severity of depressive symptoms in individuals. The CESD-8 scale is useful in clinical practice for quickly identifying potential depression patients, providing a basis for subsequent detailed assessments and treatments (17). In the research field, it also serves as an efficient tool for epidemiological studies of depressive symptoms.

The CFPS 2020 used the 8-item version of the CES-D, a simplified self-assessment scale that includes eight questions from the original CES-D scale, such as “I felt depressed,” “I felt that everything I did was an effort,” “My sleep was restless,” “I was happy,” “I felt lonely,” “I enjoyed life,” “I felt sad,” and “I could not get going.” This allows for the rapid screening of depressive symptoms. Despite the reduced number of items, the CESD-8 still covers the core dimensions of depressive mood, cognitive/psychological state, and somatic symptoms, ensuring comprehensive assessment. Similar to the original CES-D, each item on the CESD-8 is scored based on the frequency of symptoms, ranging from “rarely or none of the time” (0 points) to “most or all of the time” (3 points), with a total score range of 0 to 24 points. Higher scores indicate a higher risk of depression, with scores ≥9 indicating clinically significant depressive symptoms (18). We employed hierarchical logistic regression with interaction terms to assess effect modification by baseline/demographic factors on RDCS-depression associations [see (**Table 1**) for full results].

**Table 1:** Stratified analysis and interaction effects between RDCS and depression.

Variables	Decrease in sleep duration		*p for interaction*	Long catch-up sleep duration		*P for interaction*
	(RDCS < 0 h)	*P value*		(RDCS ≥ 2 h)	*P value*	
Age, n (%)			*0.624*			*0.996*
40≤	1.96 (1.16 – 3.30)	***0.011***		1.48 (1.21 – 1.80)	***<.001***	
> 40	1.45 (0.49 – 4.28)	*0.496*		1.47 (0.85 – 2.56)	*0.168*	
Gender, n (%)			*0.969*			*0.916*
female	1.88 (0.94 – 3.76)	*0.076*		1.49 (1.14 – 1.94)	***0.003***	
male	1.84 (0.98 – 3.47)	*0.059*		1.46 (1.13 – 1.90)	***0.004***	
Region, n (%)			*0.745*			*0.718*
countryside	1.92 (1.07 – 3.42)	***0.028***		1.52 (1.19 – 1.94)	***<.001***	
city	1.63 (0.73 – 3.61)	*0.233*		1.42 (1.06 – 1.89)	***0.017***	
Level of education n (%)			*0.810*			*0.197*
illiterate	10279892.69 (0.00 – Inf)	*0.995*		0.42 (0.05 – 3.24)	*0.403*	
elementary school	3.26 (0.41 – 25.73)	*0.263*		0.61 (0.23 – 1.62)	*0.320*	
Junior high school	1.65 (0.73 – 3.72)	*0.225*		1.57 (1.04 – 2.36)	***0.031***	
high school and above	1.79 (0.98 – 3.27)	*0.058*		1.56 (1.26 – 1.93)	***<.001***	
Marital status, n (%)			*0.197*			*0.344*
single	1.28 (0.63 – 2.59)	*0.500*		1.63 (1.22 – 2.18)	***<.001***	
married	2.38 (1.27 – 4.44)	***0.007***		1.36 (1.07 – 1.73)	***0.013***	
BMI, n (%)			*0.018*			*0.866*
<25 kg/m2	2.77 (1.49 – 5.14)	***0.001***		1.51 (1.22 – 1.87)	***<.001***	
25 to <30 kg/m2	0.66 (0.31 – 1.43)	*0.290*		1.46 (0.96 – 2.21)	*0.074*	
≥30 kg/m2	3599812.98 (0.00 – Inf)	*0.993*		1.16 (0.45 – 2.99)	*0.758*	
Health self-assessment, n (%)			*0.557*			*0.038*
bad	1.63 (0.85 – 3.12)	*0.139*		1.25 (0.97 – 1.63)	*0.089*	
fair	2.38 (1.10 – 5.18)	***0.028***		1.98 (1.48 – 2.64)	***<.001***	
good	0.98 (0.22 – 4.39)	*0.979*		0.97 (0.44 – 2.16)	*0.945*	
Chronic disease, n (%)			*0.397*			*0.346*
No	1.93 (1.18 – 3.15)	***0.009***		1.52 (1.26 – 1.84)	***<.001***	
yes	0.97 (0.21 – 4.42)	*0.965*		1.00 (0.42 – 2.35)	*0.994*	
Frequency of physical activity, n (%)			*0.293*			*0.571*
0 times a week	2.28 (1.22 – 4.26)	***0.010***		1.45 (1.15 – 1.83)	***0.002***	
1–2 times a week	1.30 (0.44 – 3.78)	*0.635*		1.55 (1.01 – 2.38)	***0.043***	
3–4 times a week	0.98 (0.28 – 3.42)	*0.969*		2.02 (0.97 – 4.23)	*0.061*	
5–6 times a week	0.12 (0.01 – 2.22)	*0.156*		0.42 (0.08 – 2.15)	*0.299*	
every day	3.50 (0.45 – 27.26)	*0.231*		1.45 (0.79 – 2.66)	*0.236*	
Smoking, n (%)			*0.801*			*0.979*
no	1.79 (1.04 – 3.08)	***0.035***		1.49 (1.20 – 1.86)	***<.001***	
yes	2.06 (0.81 – 5.24)	*0.130*		1.49 (1.03 – 2.14)	***0.032***	
Drink, n (%)			*0.623*			*0.324*
no	1.78 (1.08 – 2.91)	***0.023***		1.53 (1.26 – 1.86)	***<.001***	
yes	2.62 (0.60 – 11.40)	*0.199*		1.10 (0.59 – 2.05)	*0.758*	
Weekday sleep duration, n (%)			*0.147*			*0.681*
7h<	0.37 (0.04 – 3.59)	*0.389*		1.58 (0.94 – 2.66)	*0.086*	
≥7h	2.06 (1.27 – 3.32)	***0.003***		1.40 (1.15 – 1.72)	***<.001***	

OR, odds ratio; CI, confidence interval; RDCS, Rest day catch-up sleep.Bolded coefficients show significant interaction effects (P interaction < 0.05).

However, the reported depression prevalence (84.4%) in this study is substantially higher than global epidemiological estimates, necessitating further validation. Given that the CESD-8 is a screening tool rather than a formal diagnostic instrument, the observed prevalence may reflect subclinical depressive symptoms rather than confirmed clinical cases. Misclassification risks should be considered, particularly if symptom severity thresholds are not rigorously defined. Additionally, data entry errors or operational inconsistencies in symptom classification may have contributed to prevalence inflation. A thorough reevaluation of coding procedures, scoring criteria, and classification methods is essential to ensure data accuracy and comparability with existing epidemiological benchmarks.

This study assessed the reliability of the CESD-8 scale and obtained a Cronbach’s alpha coefficient exceeding 0.7, confirming strong internal consistency and measurement stability. This reliability metric reinforces the scale’s suitability for evaluating depressive symptoms, though refinements in classification thresholds may be necessary to enhance diagnostic precision in epidemiological research.

### 2.3 Assessments of sleep duration and RDCS

During the 2020 CFPS survey, participants responded to the following sleep-related questions to determine their sleep duration on rest days and workdays: “Excluding nap time, how many hours do you usually sleep per day on workdays?” and “How many hours do you usually sleep per day on rest days?” Here, workdays and rest days are defined by the respondents themselves, rather than by the calendar’s weekdays and weekends.

RDCS is defined as the difference in sleep duration between workdays and rest days. It is then categorized into five groups: no change in sleep duration (= 0 hours), reduced sleep duration (< 0 hours), moderate catch-up sleep duration (> 1 hour, < 2 hours), and long catch-up sleep duration (≥ 2 hours).

In current sleep duration research, data based on participants’ self-reports, despite certain limitations, are still considered to have a certain degree of accuracy and can therefore be used as research tools. This data collection method is widely adopted in the academic field and provides a reliable basis for understanding the relationship between sleep habits and health outcomes to some extent (19, 20). This study analyzes data based on participants’ self-reported information.

### 2.4 Covariates

\The analysis included a set of potential confounding variables:

1. Sociodemographic factors: age (categorized as ≤40 years or >40 years), gender (male/female), region (rural/urban), education level (illiterate/primary school/middle school/high school and above), and marital status (single/married);
2. Lifestyle factors: smoking status (yes/no), alcohol consumption (yes/no), weekday sleep duration (<7 h/≥7 h), and physical activity frequency (0/1-2/3-4/5–6 times per week or daily);
3. Health status indicators: body mass index (BMI, calculated as weight in kilograms divided by height in meters squared; categorized as ≤25 kg/m², 25–30 kg/m², or >30 kg/m²), self-rated health (assessed via 3-point Likert scale: bad/fair/good), and chronic disease status (no/yes). The chronic disease variable was derived from self-reported physician diagnoses in the CFPS survey. Although the 2020 dataset lacked specific disease classifications, prior CFPS waves confirmed inclusion of prevalent conditions such as hypertension, diabetes mellitus, and cardiovascular diseases.

### 2.5 Statistical analyses

Statistical analyses were conducted using R 4.2 software. Qualitative data were expressed as frequencies and percentages (%), and group comparisons were performed using the χ² test or Fisher’s exact test, depending on expected cell counts. Quantitative data conforming to a normal distribution were presented as mean ± standard deviation (x ± s), and between-group comparisons were conducted using the t-test. To assess the relationship between RDCS and depressive symptoms, both univariate logistic analysis and multivariate logistic regression models were employed. Three models were established: Model 1: unadjusted; Model 2: adjusted for age, gender, education level, marital status, and region; Model 3: further adjusted for age, gender, education level, marital status, region, BMI, health self-assessment, smoking status, alcohol consumption, presence of chronic diseases, frequency of physical activity, and weekday sleep duration. Additionally, stratified logistic regression analysis was performed to examine whether the association between RDCS and depression varied across subgroups defined by demographic or health-related factors, ensuring a robust assessment of population differences. The hierarchical structure of the stratified model was defined as follows: Level 1: Core demographic variables (age, gender, region, education level, marital status); Level 2: Lifestyle factors (smoking, drinking, physical activity frequency, weekday sleep duration); Level 3: Health status indicators (BMI, self-rated health, chronic disease status). To maintain consistency in comparison, reference groups were designated as follows: RDCS groups: No change in sleep duration (RDCS = 0 h) as the baseline category; Weekday sleep duration: ≥7 hours as the baseline reference; BMI: <25 kg/m² as the reference group. Stratification variables (BMI and self-rated health) were selected based on prior studies indicating their moderating effects on the sleep-depression relationship. Additionally, interaction terms (e.g., RDCS × BMI) were tested to validate the robustness of stratification in capturing subgroup-specific effects. All statistical tests were two-tailed, with P < 0.05 considered statistically significant.

## 3 Results

### 3.1 Participant characteristics

This study included 3,981 participants. **Table 2** presents the characteristics of the subjects (no change in sleep duration (RDCS = 0 h), reduced sleep duration (RDCS < 0), moderate catch-up sleep duration (0 h < RDCS < 2 h), and long catch-up sleep duration (RDCS ≥ 2 h)). Compared to other groups, the short catch-up sleep group had the lowest proportion of participants with depression, while the reduced sleep duration group had the highest proportion of participants with depression. The reduced sleep duration group also had the least sleep on rest days and the longest sleep duration on workdays.

**Table 2:** Baseline characteristics of participants (n=3,981).

Characteristics	Total	RDCS groups				*p-value*
		No alteration in duration	Decrease in sleep duration	Moderate catch-up sleep duration	Long catch-up sleep duration	
		(RDCS=0h)	(RDCS<0h)	(0 h < RDCS < 2 h)	(RDCS ≥ 2 h)	
	n = 3981	n = 1291	n = 215	n = 999	n = 1476	
Age, Mean ± SD	30.46 ± 7.11	31.88 ± 6.93	31.23 ± 7.35	30.58 ± 7.17	29.04 ± 6.92	***<.001***
Median age (IQR)	30 (25-35)	32 (27-36)	31 (26-36)	30 (25-35)	28(24-33)	***<.001***
Weekday sleep duration, Mean ± SD	7.51 ± 1.11	7.78 ± 0.97	8.60 ± 1.36	7.36 ± 0.86	7.22 ± 1.19	***<.001***
Rest day sleep duration, Mean ± SD	8.67 ± 1.62	7.78 ± 0.97	6.86 ± 1.39	8.37 ± 0.87	9.93 ± 1.62	***<.001***
CESD-8, Mean ± SD	13.03 ± 3.52	12.65 ± 3.25	13.86 ± 3.90	12.83 ± 3.36	13.37 ± 3.74	***<.001***
Age, n (%)						*<.001*
40≤	3486 (87.57)	1089 (84.35)	185 (86.05)	871 (87.19)	1341 (90.85)	
> 40	495 (12.43)	202 (15.65)	30 (13.95)	128 (12.81)	135 (9.15)	
Gender, n (%)						*0.001*
female	1976 (49.64)	590 (45.70)	105 (48.84)	489 (48.95)	792 (53.66)	
male	2005 (50.36)	701 (54.30)	110 (51.16)	510 (51.05)	684 (46.34)	
Region, n (%)						*<.001*
countryside	2386 (59.93)	742 (57.47)	154 (71.63)	562 (56.26)	928 (62.87)	
city	1595 (40.07)	549 (42.53)	61 (28.37)	437 (43.74)	548 (37.13)	
Level of education n (%)						*<.001*
illiterate	55 (1.38)	28 (2.17)	6 (2.79)	4 (0.40)	17 (1.15)	
elementary school	156 (3.92)	59 (4.57)	22 (10.23)	25 (2.50)	50 (3.39)	
Junior high school	747 (18.78)	245 (18.99)	61 (28.37)	150 (15.02)	291 (19.74)	
high school and above	3020 (75.92)	958 (74.26)	126 (58.60)	820 (82.08)	1116 (75.71)	
Marital status, n (%)						*<.001*
single	1641 (41.22)	435 (33.69)	77 (35.81)	414 (41.44)	715 (48.44)	
married	2340 (58.78)	856 (66.31)	138 (64.19)	585 (58.56)	761 (51.56)	
BMI, n (%)						*0.546*
<25 kg/m^2^	3096 (77.79)	991 (76.76)	174 (80.93)	775 (77.58)	1156 (78.37)	
25 to <30 kg/m^2^	759 (19.07)	256 (19.83)	39 (18.14)	192 (19.22)	272 (18.44)	
≥30 kg/m^2^	125 (3.14)	44 (3.41)	2 (0.93)	32 (3.20)	47 (3.19)	
Health self-assessment, n (%)						*0.499*
bad	1566 (39.34)	516 (39.97)	81 (37.67)	375 (37.54)	594 (40.24)	
fair	2061 (51.77)	665 (51.51)	110 (51.16)	524 (52.45)	762 (51.63)	
good	354 (8.89)	110 (8.52)	24 (11.16)	100 (10.01)	120 (8.13)	
Chronic disease, n (%)						*0.349*
No	3711 (93.22)	1207 (93.49)	194 (90.23)	931 (93.19)	1379 (93.43)	
yes	270 (6.78)	84 (6.51)	21 (9.77)	68 (6.81)	97 (6.57)	
Frequency of physical activity, n (%)						*<.001*
0 times a week	2493 (62.62)	808 (62.59)	146 (67.91)	569 (56.96)	970 (65.72)	
1~2 times a week	746 (18.74)	228 (17.66)	30 (13.95)	222 (22.22)	266 (18.02)	
3~4 times a week	360 (9.04)	113 (8.75)	23 (10.70)	101 (10.11)	123 (8.33)	
5~6 times a week	57 (1.43)	20 (1.55)	2 (0.93)	20 (2.00)	15 (1.02)	
every day	325 (8.16)	122 (9.45)	14 (6.51)	87 (8.71)	102 (6.91)	
Smoking, n (%)						*0.001*
no	3026 (76.01)	973 (75.37)	159 (73.95)	804 (80.48)	1090 (73.85)	
yes	955 (23.99)	318 (24.63)	56 (26.05)	195 (19.52)	386 (26.15)	
Drink, n(%)						***0.008***
no	3655 (91.81)	1176 (91.09)	187 (86.98)	935 (93.59)	1357 (91.94)	
yes	326 (8.19)	115 (8.91)	28 (13.02)	64 (6.41)	119 (8.06)	
Weekday sleep duration, n (%)						*<.001*
7h<	581 (14.59)	107 (8.29)	4 (1.86)	139 (13.91)	331 (22.43)	
≥7h	3400 (85.41)	1184 (91.71)	211 (98.14)	860 (86.09)	1145 (77.57)	
Depression, n (%)						*<.001*
No	622 (15.62)	247 (19.13)	20 (9.30)	171 (17.12)	184 (12.47)	
yes	3359 (84.38)	1044 (80.87)	195 (90.70)	828 (82.88)	1292 (87.53)	

RDCS, Rest day catch-up sleep.Bold values indicate statistically significant associations (P < 0.05).

### 3.2 Univariate analysis of the association between depression with covariates

**Table 3** uses univariate regression analysis to observe the association between age, gender, region, education level, BMI, smoking, drinking, marital status, health self-assessment, chronic disease, frequency of physical activity, weekday sleep duration, RDCS, and depression.

**Table 3:** Univariate analysis of the association between depression with covariates.

Variables	*P*	OR (95%CI)
Age, n (%)		
40≤		1.00 (Reference)
> 40	0.123	0.82 (0.64 – 1.05)
Gender, n (%)		
female		1.00 (Reference)
male	0.085	0.86 (0.72 – 1.02)
Region, n (%)		
countryside		1.00 (Reference)
city	**0.027**	0.82 (0.69 – 0.98)
Level of education n (%)		
illiterate		1.00 (Reference)
elementary school	0.320	0.57 (0.18 –1.74)
Junior high school	0.066	0.38 (0.13 – 1.07)
high school and above	0.100	0.42 (0.15 – 1.18)
Marital status, n (%)		
single		1.00 (Reference)
married	0.058	0.84 (0.71 – 1.01)
BMI, n (%)		
<25 kg/m2		1.00 (Reference)
25 to <30 kg/m2	0.261	0.88 (0.71 – 1.10)
≥30 kg/m2	0.333	0.80 (0.50 – 1.26)
Health self-assessment, n (%)		
bad		1.00 (Reference)
fair	**<.001**	1.60 (1.34 – 1.91)
good	**<.001**	2.81 (1.89 –4.19)
Chronic disease, n (%)		
No		1.00 (Reference)
yes	**0.003**	1.88 (1.23 – 2.86)
Frequency of physical activity, n (%)		
0 times a week		1.00 (Reference)
1~2 times a week	0.395	0.91 (0.73 – 1.13)
3~4 times a week	0.228	1.22 (0.88 – 1.70)
5~6 times a week	0.544	1.28 (0.58 – 2.85)
every day	**0.012**	0.69 (0.52 – 0.92)
Smoking, n (%)		
no		1.00 (Reference)
yes	0.488	0.93 (0.76 – 1.14)
Drink, n (%)		
no		1.00 (Reference)
yes	0.865	0.97 (0.71 – 1.33)
Weekday sleep duration, n (%)		
7h<		1.00 (Reference)
≥7h	**0.001**	0.63 (0.48 – 0.83)
RDCS duration		
No alteration in sleep duration (RDCS = 0 h)		1.00 (Reference)
Decrease in sleep duration (RDCS < 0 h)	**<.001**	2.31 (1.43 – 3.73)
Moderate catch-up duration (1 h < RDCS < 2 h)	0.216	1.15 (0.92 – 1.42)
Long catch-up duration (RDCS ≥ 2 h)	**<.001**	1.66 (1.35 – 2.04)

OR, odds ratio; CI, confidence interval; RDCS, Rest day catch-up sleep.Bold values represent significant adjusted odds ratios (95% CI not containing 1.0).

Regarding region, compared to rural areas, urban participants were less likely to have depression (OR =0.82, 95%CI [0.69 – 0.98]). In terms of health self-assessment, participants with a fair (OR= 1.60, 95%CI [1.34 – 1.91]) or good (OR =2.81, 95%CI [1.89 – 4.19]) self-assessment were more likely to have depression compared to those with a poor self-assessment. Participants with chronic diseases were more likely to have depression compared to those without chronic diseases (OR= 1.88, 95%CI [1.23 – 2.86]). Participants with weekday sleep duration ≥7 hours were less likely to have depression compared to those with weekday sleep duration <7 hours (OR =0.63, 95%CI [0.48 – 0.83]). Compared to participants with no change in rest day sleep duration, those with decreased sleep duration (OR =2.31, 95%CI [1.43 – 3.73]) or long catch-up sleep duration (OR= 1.66, 95%CI [1.35 – 2.04]) had significantly increased rates of depression.

### 3.3 Multivariate logistic regression analysis of the relationship between RDCS and depression

We established three multivariable logistic regression models, as shown in **Table 4**, to analyze the relationship between RDCS and depression: Model 1, unadjusted; Model 2, adjusted for age, gender, level of education, marital status, and region; Model 3, adjusted for age, gender, level of education, marital status, region, BMI, health self-assessment, smoking, drinking, chronic disease, frequency of physical activity, and weekday sleep duration.

**Table 4:** Multivariate logistic regression analysis of the association between RDCS and depressive symptoms.

RDCS duration	Model 1		Model 2		Model 3	
	OR (95 % CI)	*P value*	OR (95 % CI)	*P value*	OR (95 % CI)	*P value*
No alteration in sleep duration (RDCS = 0 h)	ref	*ref*	ref	*ref*	ref	*ref*
Decrease in sleep duration (RDCS < 0 h)	2.31 (1.43 – 3.73)	***<.001***	2.25 (1.39 –3.65)	***<.001***	2.12 (1.31 – 3.46)	***<.002***
Moderate catch-up duration (1 h < RDCS < 2 h)	1.15 (0.92 – 1.42)	*<.00216*	1.14 (0.92 – 1.42)	*<.0230*	1.09 (0.87 – 1.36)	*<.442*
Long catch-up duration (RDCS ≥ 2 h)	1.66 (1.35 – 2.04)	***<.001***	1.61 (1.31 – 1.99)	***<.001***	1.60 (1.29 – 1.98)	***<.001***

CI, confidence interval; OR, odds ratio; RDCS, Rest day catch-up sleep.

Model 1, unadjusted.

Model 2, adjust for age, gender, level of education, marital status and region.

Model 3, adjust for age, gender, level of education, marital status and region, BMI, Health self-assessment, smoking, drink, Chronic disease, Frequency of physical activity, Weekday sleep duration.Bold type highlights primary outcome measures with P < 0.01.

In Models 1 and 2, participants with decreased sleep duration (RDCS < 0 h) (Model 1: OR =2.31, 95%CI [1.43 – 3.73], P <.001; Model 2: OR =2.25, 95%CI [1.39 – 3.65], P = 0.001) and those with long catch-up sleep duration (RDCS ≥ 2 h) (Model 1: OR= 1.66, 95%CI [1.35 – 2.04], P < 0.001; Model 2: OR =1.61, 95%CI [1.31 – 1.99], P < 0.001) showed significant associations with depression.

In Model 3, after adjusting for all covariates, participants with decreased sleep duration (RDCS < 0 h) (OR= 2.12, 95%CI [1.31 – 3.46], P = 0.002) and those with long catch-up sleep duration (RDCS ≥ 2 h) (OR= 1.60, 95%CI [1.29 – 1.98], P < 0.001) still showed statistically significant associations with depression.

### 3.4 Hierarchical analysis and interaction effects

We used stratified logistic regression analysis and interaction effect analysis to determine whether the association between RDCS and depression depends on baseline or demographic factors. The association between RDCS < 0 h and depression showed an interaction with BMI (interaction P-values were all less than 0.05), indicating that for individuals with a BMI < 25 kg/m², the likelihood of depression increases if RDCS < 0 h compared to other groups. RDCS < 0 h is only associated with the likelihood of depression in specific populations.

The association between RDCS ≥ 2 h and depression showed an interaction with health self-assessment (interaction P-values were all less than 0.05), indicating that for individuals with a general health self-assessment, the likelihood of depression increases if RDCS ≥ 2 h compared to other groups. RDCS ≥ 2 h is only associated with the likelihood of depression in specific populations.

Stratified by weekday sleep duration (<7h vs. ≥7h), the association between RDCS<0h and depression was significant only in those with adequate weekday sleep (≥7h: OR=2.06, 95%CI 1.27-3.32, p=0.003) but not in sleep-deprived individuals (<7h: OR=0.37, 95%CI 0.04-3.59, p=0.389). For RDCS≥2h, the association remained significant in both subgroups (≥7h: OR=1.40; <7h: OR=1.58) though the latter did not reach statistical significance (p=0.086), possibly due to smaller sample size.

## 4 Discussion

This study provides novel evidence regarding the association between RDCS patterns and depression among Chinese adults during the COVID-19 pandemic. Our analysis of 3,981 participants demonstrates that both reduced sleep duration (RDCS<0h) and excessive catch-up sleep (RDCS≥2h) were significantly associated with elevated depression likelihood, with adjusted odds ratios of 2.12 (95% CI 1.31-3.46) and 1.60 (95% CI 1.29-1.98) respectively in fully adjusted models (6, 7). These findings align with previous reports of U-shaped relationships between sleep duration and depression susceptibility (21, 22), while specifically highlighting the mental health impacts of irregular sleep compensation patterns during public health crises. The biological plausibility of these associations is supported by multiple pathways. For RDCS<0h, the increased depression vulnerability may involve inflammatory activation impairing serotonin synthesis through the kynurenine pathway (23, 24), coupled with reduced Brain-Derived Neurotrophic Factor (BDNF) l levels that compromise neural plasticity and emotional regulation (25). Conversely, RDCS≥2h likely induces circadian rhythm disruption through significant sleep schedule shifts (26–28), while providing incomplete compensation for accumulated sleep debt (29). Our subgroup analyses further revealed that these effects were particularly pronounced in individuals with BMI<25 kg/m² (OR=2.77) and those reporting fair self-rated health status, suggesting metabolic state and subjective health perception may influence predisposition to sleep-related mood disturbances.

Notably, the differential effects based on weekday sleep sufficiency provide important clinical insights. The particularly strong adverse impact of reduced weekend sleep (RDCS<0h) among those obtaining adequate weekday sleep (≥7h) suggests voluntary sleep restriction on rest days may be more detrimental than chronic sleep deprivation. This observation aligns with emerging evidence that acute sleep restriction has distinct neurobiological consequences compared to chronic sleep debt (29, 30). Meanwhile, the consistent association between excessive catch-up sleep (RDCS≥2h) and depression across sleep sufficiency groups, though limited by sample size in the sleep-deprived subgroup, reinforces concerns about circadian disruption from variable sleep patterns.

The observed 84.4% depression prevalence, while substantially higher than pre-pandemic estimates, can be contextualized by both methodological considerations and the unique circumstances of the pandemic. The screening nature of CESD-8, which captures subclinical symptoms rather than clinical diagnoses (17, 18), combined with the documented 27.6% global increase in depression cases during this period (14), suggests this finding reflects both measurement characteristics and genuine mental health deterioration. While our cross-sectional design precludes causal inference, these results contribute to growing evidence that maintaining stable sleep patterns may be particularly crucial for mental health during population-wide stressors.

### 4.1 Limitations

Firstly, this study is a cross-sectional study, so it cannot establish a causal relationship between sleep problems (RDCS) and depressive symptoms. Although we found some correlations, the lack of longitudinal studies prevents us from inferring causal relationships over time. Future research should collect new data post-pandemic and combine it with the 2020 data to further explore the causal relationship between the two. Due to the lack of publicly available information specifying the exact data collection timeline for CFPS 2020, we are unable to accurately differentiate sleep patterns and mental health changes across different periods of the pandemic. The absence of this temporal information may affect the precision of our analysis regarding the pandemic’s impact and could limit the applicability of certain findings across different stages. Therefore, future research should incorporate more precise time data to further explore the relationship between sleep and depression across various phases of the pandemic.

Secondly, the assessment of depressive symptoms in this study relies on standardized questionnaires (CES-D) rather than clinical psychiatric evaluations. This method may weaken the credibility of the results. Although the CES-D has been validated in the Chinese population and shows good validity, it is not the gold standard for diagnosing depression. Therefore, our study may have the risk of overestimating or underestimating depressive symptoms. Future research could consider combining clinical psychiatric evaluations to improve diagnostic accuracy.

Additionally, the collection of sleep data relies on participants’ self-reports, which may lead to recall bias. Due to the vagueness of memory, participants may not accurately report their sleep conditions, affecting the accuracy of the study results. To reduce this bias, future research could consider using objective sleep monitoring methods, such as sleep logs, actigraphy, or polysomnography. The dataset lacked information on work shifts, preventing an assessment of how shift work might influence sleep compensation patterns. Future studies should incorporate work schedule data to enable more precise analyses. Although we conducted subgroup analyses by weekday sleep duration, the relatively small sample size in the sleep-deprived subgroup (<7h) may have limited our power to detect significant associations, particularly for RDCS≥2h. The "no change in sleep duration" group (RDCS=0) did not account for minor fluctuations (e.g., ± 30 minutes), failing to consider natural variations in sleep duration. A small margin ( ± 30 minutes) might better reflect biological sleep regulation while maintaining clinical relevance. Subsequent studies could validate this relationship through more refined grouping.

A key limitation of this study is the inability to directly control for pandemic-related lifestyle changes (e.g., remote work, social isolation, and increased psychological stress) because the current analysis only adjusted for demographic variables (age, gender, region, education level, and marital status). Although these variables partially reflect the demographic background of the respondents, additional indicators such as employment status, detailed socioeconomic status, and household structure which might better capture the impact of the COVID-19 pandemic were not incorporated as separate covariates. Future studies should integrate these individual-level variables to more comprehensively assess the effects of the pandemic on sleep patterns and depression.

Lastly, this study did not fully consider other psychosocial factors that may affect the relationship between sleep problems and depressive symptoms, such as work stress and family support. These factors may play a mediating or moderating role between the two. Therefore, future research should include these psychosocial factors in the analytical framework to more comprehensively understand the relationship between sleep and depression.

Despite these limitations, we hope that these findings can provide valuable references for subsequent research and continuously improve research design in future studies to reveal the exact relationship between sleep and depression. Through these efforts, we aim to provide stronger scientific evidence for the prevention, diagnosis, and treatment of depression.

## 5 Conclusion

This study reveals the association between RDCS patterns and the likelihood of depression during the pandemic. The results indicate that both reduced sleep duration and excessive catch-up sleep may lead to an increased likelihood of depression. These findings suggest that maintaining regular sleep habits is crucial for mental health, even during the pandemic. Additionally, the results show that BMI and health self-assessment may be important factors influencing the relationship between sleep compensation and the likelihood of depression. Therefore, public health interventions should consider these demographic characteristics to more effectively prevent and mitigate the likelihood of depression. Future research can further explore the potential biological mechanisms of sleep compensation and how to develop personalized sleep intervention strategies for different populations.

## Data Availability

The datasets presented in this study can be found in online repositories. The acquisition of the data is a public database, and the link is: https://www.isss.pku.edu.cn/cfps/sjzx/gksj/index.htm.

## References

[1] Herrman H Kieling C McGorry P Horton R Sargent J Patel V. Reducing the global burden of depression: a Lancet-World Psychiatric Association Commission. Lancet. (2019) 393:e42–3. doi: 10.1016/S0140-6736(18)32408-5 30482607

[2] Malhi GS Mann JJ. Depression. Lancet. (2018) 392:2299–312. doi: 10.1016/S0140-6736(18)31948-2 30396512

[3] Bayram N Bilgel N. The prevalence and socio-demographic correlations of depression, anxiety and stress among a group of university students. Soc Psychiatry Psychiatr Epidemiol. (2008) 43:667–72. doi: 10.1007/s00127-008-0345-x 18398558

[4] Hamilton OS Steptoe A Ajnakina O. Polygenic predisposition, sleep duration, and depression: evidence from a prospective population-based cohort. Transl Psychiatry. (2023) 13:323. doi: 10.1038/s41398-023-02622-z 37857612 PMC10587060

[5] Palagini L Baglioni C Ciapparelli A Gemignani A Riemann D. REM sleep dysregulation in depression: state of the art. Sleep Med Rev. (2013) 17:377–90. doi: 10.1016/j.smrv.2012.11.001 23391633

[6] Li XL Wei J Zhang X Meng Z Zhu W. Relationship between night-sleep duration and risk for depression among middle-aged and older people: A dose-response meta-analysis. Front Physiol. (2023) 2(4):1085091. doi: 10.3389/fphys.2023.1085091 PMC10017495 36935736

[7] Dong L Xie Y Zou X. Association between sleep duration and depression in US adults: A cross-sectional study. J Affect Disord. (2022) 296:183–8. doi: 10.1016/j.jad.2021.09.075 34607059

[8] Ford ES TJ C Croft JB. Trends in self-reported sleep duration among US adults from 1985 to 2012. Sleep. (2015) 38:829–32. doi: 10.5665/sleep.4684 PMC4402659 25669182

[9] Akbar SA Mattfeld AT Laird AR McMakin DL. Sleep to Internalizing Pathway in Young Adolescents (SIPYA): A proposed neurodevelopmental model. Neurosci Biobehav Rev. (2022) 140:104780. doi: 10.1016/j.neubiorev.2022.104780 35843345 PMC10750488

[10] Son SM EJ P YH C SY L JI C YI L. Association between weekend catch-up sleep and metabolic syndrome with sleep restriction in korean adults: A cross-sectional study using KNHANES. Diabetes Metab Syndr Obes. (2020) 13:1465–71. doi: 10.2147/DMSO.S247898 PMC7200717 32431530

[11] Zhu H Qin S Wu M. Association between weekend catch-up sleep and cardiovascular disease: Evidence from the National Health and Nutrition Examination Surveys 2017-2018. Sleep Health. (2024) 10:98–103. doi: 10.1016/j.sleh.2023.09.006 38000943

[12] Oh J Kim E Huh I. Associations between weekend catch-up sleep and health-related quality of life with focusing on gender differences. Sci Rep. (2023) 13:20280. doi: 10.1038/s41598-023-47244-z 37985799 PMC10662263

[13] Fernández-Alcántara M CK K-K Cruz-Quintana F Pérez-Marfil MN. Editorial: new perspectives in bereavement and loss: complicated and disenfranchised grief along the life cycle. Front Psychol. (2021) 12:691464. doi: 10.3389/fpsyg.2021.691464 34113302 PMC8185040

[14] Santomauro DF Mantilla Herrera AM Shadid J Zheng P Ashbaugh C Pigott DM. Global prevalence and burden of depressive and anxiety disorders in 204 countries and territories in 2020 due to the COVID-19 pandemic. Lancet. (2021) 398:1700–12. doi: 10.1016/S0140-6736(21)02143-7 PMC8500697 34634250

[15] Jahrami H AS B NL B Saif Z Faris M Vitiello MV. Sleep problems during the COVID-19 pandemic by population: a systematic review and meta-analysis. J Clin Sleep Med. (2021) 17:299–313. doi: 10.5664/jcsm.8930 33108269 PMC7853219

[16] Radloff LS. The CES-D scale A self-report depression scale for research in the general population. Appl psychol Measurement. (1977) 1:385–401. doi: 10.1177/014662167700100306

[17] Briggs R Carey D AM O RA K Kennelly SP. Validation of the 8-item Centre for Epidemiological Studies Depression Scale in a cohort of community-dwelling older people: data from The Irish Longitudinal Study on Ageing (TILDA). Eur Geriatr Med. (2018) 9:121–6. doi: 10.1007/s41999-017-0016-0 34654281

[18] Wu Y Su B Chen C Zhao Y Zhong P Zheng X. Urban-rural disparities in the prevalence and trends of depressive symptoms among Chinese elderly and their associated factors. J Affect Disord. (2023) 340:258–68. doi: 10.1016/j.jad.2023.07.117 37536424

[19] Smiley A King D Bidulescu A. The association between sleep duration and metabolic syndrome: the NHANES 2013/2014. Nutrients. (2019) 11:2582. doi: 10.3390/nu11112582 31717770 PMC6893635

[20] Wang S ME R RR N Nguyen US. Interaction between sleep duration and trouble sleeping on depressive symptoms among U.S. adults, NHANES 2015-2018. J Affect Disord. (2024) 351:285–92. doi: 10.1016/j.jad.2024.01.260 38302062

[21] Liu BP XT W ZZ L ZY W An D YX W. Depressive symptoms are associated with short and long sleep duration: A longitudinal study of Chinese adolescents. J Affect Disord. (2020) 263:267–73. doi: 10.1016/j.jad.2019.11.113 31818788

[22] Zhai L Zhang H Zhang D. SLEEP DURATION AND DEPRESSION AMONG ADULTS: A META-ANALYSIS OF PROSPECTIVE STUDIES. Depress Anxiety. (2015) 32:664–70. doi: 10.1002/da.22386 26047492

[23] Prather AA Vogelzangs N Penninx BW. Sleep duration, insomnia, and markers of systemic inflammation: results from the Netherlands Study of Depression and Anxiety (NESDA). J Psychiatr Res. (2015) 60:95–102. doi: 10.1016/j.jpsychires.2014.09.018 25307717 PMC4250403

[24] Hall MH SF S RM B HN A SE G TB H. Association between sleep duration and mortality is mediated by markers of inflammation and health in older adults: the Health, Aging and Body Composition Study. Sleep. (2015) 38:189–95. doi: 10.5665/sleep.4394 PMC4288599 25348127

[25] Rahmani M Rahmani F Rezaei N. The brain-derived neurotrophic factor: missing link between sleep deprivation, insomnia, and depression. Neurochem Res. (2020) 45:221–31. doi: 10.1007/s11064-019-02914-1 31782101

[26] Islam Z Akter S Kochi T Hu H Eguchi M Yamaguchi M. Association of social jetlag with metabolic syndrome among Japanese working population: the Furukawa Nutrition and Health Study. Sleep Med. (2018) 51:53–8. doi: 10.1016/j.sleep.2018.07.003 30099352

[27] Kantermann T Duboutay F Haubruge D Kerkhofs M Schmidt-Trucksäss A Skene DJ. Atherosclerotic risk and social jetlag in rotating shift-workers: first evidence from a pilot study. Work. (2013) 46:273–82. doi: 10.3233/WOR-121531 23324695

[28] Parsons MJ TE M AM G Goldman-Mellor S PM N Poulton R. Social jetlag, obesity and metabolic disorder: investigation in a cohort study. Int J Obes (Lond). (2015) 39:842–8. doi: 10.1038/ijo.2014.201 PMC4422765 25601363

[29] Depner CM EL M RH E JK S-B Perreault L BC B. Ad libitum Weekend Recovery Sleep Fails to Prevent Metabolic Dysregulation during a Repeating Pattern of Insufficient Sleep and Weekend Recovery Sleep. Curr Biol. (2019) 29:957–967.e4. doi: 10.1016/j.cub.2019.01.069 30827911 PMC12798825

[30] Liu Y Yin J Li X Yang J Liu Y. Examining the connection between weekend catch-up sleep and depression: Insights from 2017 to 2020 NHANES information. J Affect Disord. (2024) 358:61–9. doi: 10.1016/j.jad.2024.05.022 38705524

